# An optimized method for mulberry silkworm, *Bombyx mori* (Bombycidae:Lepidoptera) sex classification using TLBPSGA-RFEXGBoost

**DOI:** 10.1242/bio.060468

**Published:** 2024-07-18

**Authors:** Sania Thomas, Jyothi Thomas

**Affiliations:** Department of Computer Science and Engineering, Christ University, Bangalore, 560029, India

**Keywords:** Sericulture, Accelerated HOG, RFEXGBoost, TLBPSGA, Seed production, Classification

## Abstract

Silkworm seed production is vital for silk farming, requiring precise breeding techniques to optimize yields. In silkworm seed production, precise sex classification is crucial for optimizing breeding and boosting silk yields. A non-destructive approach for sex classification addresses these challenges, offering an efficient alternative that enhances both yield and environmental responsibility. Southern India is a hub for mulberry silk and cocoon farming, with the high-yielding double-hybrid varieties FC1 (foundation cross 1) and FC2 (foundation cross 2) being popular. Traditional methods of silkworm pupae sex classification involve manual sorting by experts, necessitating the cutting of cocoons – a practice with a high risk of damaging the cocoon and affecting yield. To address this issue, this study introduces an accelerated histogram of oriented gradients (HOG) feature extraction technique that is enhanced by block-level dimensionality reduction. This non-destructive method allows for efficient and accurate silkworm pupae classification. The modified HOG features are then fused with weight features and processed through a machine learning classification model that incorporates recursive feature elimination (RFE). Performance evaluation shows that an RFE-hybridized XGBoost model attained the highest classification accuracy, achieving 97.2% for FC1 and 97.1% for FC2. The model further optimized with a novel teaching learning-based population selection genetic algorithm (TLBPSGA) achieved a remarkable accuracy of 98.5% for FC1 and 98.2% for FC2. These findings have far-reaching implications for improving both the ecological sustainability and economic efficiency of silkworm seed production.

## Teaser

**Summary:** This study introduces an optimized non-destructive silkworm pupae sex classification method that enhances both ecological sustainability and economic efficiency in silkworm seed production.

## INTRODUCTION

Sericulture is a complex process of cultivating silkworms for the production of silk that requires careful attention to detail and a deep understanding of the life cycle of the silkworm. The different steps involved in sericulture includes mulberry cultivation, egg production, silkworm rearing, cocoon production, harvesting, and weaving (Altman and Farrell, 2022; Gundi et al., 2023; Thomas and Thomas, 2020). The advancement of silkworm breeding is crucial for high quality silk and cocoon production. A significant breakthrough in this field was the introduction of the CSR2×CSR4 hybrid breed in India in 1997, transforming the country's sericulture industry. Alongside this, the CSR hybrids, specifically FC1 (CSR6×CSR26) and FC2 (CSR2×CSR27), have proven to be highly productive and robust, suitable for cultivation by farmers. These breeds, however, require meticulous care to ensure the availability of parental cocoons. To mitigate the challenges associated with these pure breeds, bivoltine double hybrid breeds have been developed. These hybrids are more resistant to adverse weather conditions, resulting in more consistent crop yields compared to their single hybrid counterparts. One such successful double hybrid breed in India is (CSR2×CSR27)×(CSR6×CSR26), capable of yielding around 68.00 kg of silk per 100 disease-free layings (dfls). In our study we used single hybrid FC1 and FC2 varieties. Egg production is one of the key steps in sericulture that involves obtaining healthy silkworm eggs for the next generation of silkworms. Egg production involves the selection of parent stock, mating, egg laying, incubation, harvesting the eggs and storage. Selecting healthy parent stock is a crucial phase in egg production. In the traditional method, the cocoons are cut open, and the pupae are visually inspected to determine their sex. The traditional method is a destructive method that has several disadvantages, such as: damage to the cocoon; risk of bacterial and fungal infections in the pupa, which can lead to lower survival rates and reduced silk quality; cutting open the cocoon can be stressful and traumatic for the pupa, leading to a higher mortality rate among the pupae; the traditional method is a time-consuming and labour intensive process, requiring skilled workers to carefully handle the delicate silkworm pupa; and, the accuracy of sex classification by visual inspection can vary depending on the skill and experience of the worker performing the classification, leading to inconsistencies and errors in the final product. Introduction of non-destructive methods for sex classification of silkworm pupae has the following advantages such as: maintaining cocoon quality, reducing the risk of infection, and saves time and labour. The non-destructive method makes the overall sericulture process more efficient and cost-effective.

There are several available methods; polymerase chain reaction (PCR) is a molecular biology technique that can be used to amplify and detect specific DNA (deoxyribonucleic acid) sequences (Reddy et al., 1999). Sex-specific markers have been identified in silkworms, which can be used to identify the sex of the pupa by analyzing its DNA. This method is highly accurate and reliable but requires specialized equipment and trained personnel. Near infrared spectroscopy (NIR) is a method that uses light to analyze the chemical composition of a substance. It has been used to identify the sex of silkworm pupae based on differences in their chemical composition (Zhu et al., 2018; Fu et al., 2023; Lin et al., 2019; Tao et al., 2018; Qiu et al., 2021). In the NIR method, environmental factors can affect the accuracy, and the instrument requires calibration and expertise, which is expensive.

Sex specific dyes are a non-destructive method (Traut et al., 2008). In this method certain dyes have been developed that are specific to male or female silkworm pupae. These dyes are injected into the pupae, and their coloration indicates their sex. There are some problems that can limit their widespread use, including that some sex-specific dyes may be toxic to silkworm pupae, which can reduce the survival rate and overall health of the pupae. The accuracy of sex-specific dyes can vary depending on factors such as the type and concentration of dye used, as well as environmental conditions. This can lead to inconsistencies in the sex classification of pupae. Sex-specific dyes may have a limited shelf life, meaning they need to be stored and used within a certain time frame. Sex-specific dyes need to be applied carefully and accurately to the pupae, which can be a time-consuming and labor-intensive process. This can increase the cost of production. The use of sex-specific dyes can increase the risk of contamination in the production process.

X-ray imaging techniques were also experimented with in the classification of silkworm pupae without cutting the cocoon (Cai et al., 2014). In previous research, we explored the use of X-ray imaging as a non-destructive technique for the sex classification of silkworm pupae. By employing X-ray imaging, we were able to investigate the internal structures of the pupae without damaging the cocoon. Although this approach holds promise for more precise and non-invasive classification, two major challenges identified were the financial burden associated with the acquisition and operation of specialized X-ray equipment, and concerns about the safety of exposing silkworm pupae to ionizing radiation. Despite these hurdles, our study serves as a pioneering effort in employing advanced imaging technology for the enhancement of sericulture practices (Thomas and Thomas, 2022).

A camera imaging method for pupae sex classification in silkworms is becoming increasingly popular in the sericulture industry. Frontal gonad images are utilized in the classification process (Guo et al., 2022). For this purpose, Guo et al. developed an image exposure correction algorithm to address underexposure and overexposure issues in the images. This approach achieved an accuracy of 90.5%, which was approximately 7% higher than using the original gonad images. He et al. (2023) presents a comprehensive approach for identifying silkworm pupae species and sex using machine learning and deep learning techniques using pupae images. This method obtained an accuracy of 99% for sex classification. The camera images of the cocoon are also used for the sex classification, which eliminates cutting the cocoon. Shape features of the cocoon is studied for the classification process (Joseph Raj et al., 2019; Mahesh et al., 2017). Mahesh et al. (2017) used various combinations of weight, volume, geometric, and Zernike moment-based shape features for discrimination. A mean classification accuracy of 91.3% was attained with the neural network for CSR2 and 100% with the support vector machine (SVM) for the Pure Mysore breed. Joseph Raj et al. (2019) used the combination of weight data with shape features to differentiate male and female pupae and attained an accuracy ranging from 86.48% to 93.54% using SVM. Our previous study explored different feature extraction method such as Zernike moment-based features, Gabor feature, gray level co-occurrence matrix (GLCM), local binary pattern (LBP), and histogram of oriented gradients (HOG). The study identified HOG features best suitable for our problem (Thomas and Thomas, 2024). HOG-based descriptors are a widely used technique in image classification. They capture the distribution of gradient orientations in an image, providing a robust representation that is particularly effective in detecting object shapes and textures (Nam et al., 2011; Klein and Celik, 2017). The HOG feature descriptor is used in many areas, including gender recognition from face images, object and pedestrian localization, attentive semantic alignment, emotion recognition and personality analysis (Son et al., 2015; Yavariabdi et al., 2022; Seo et al., 2018; Yavariabdi et al., 2021; Nam et al., 2014; Moghimi et al., 2022). In our proposed method, accelerated HOG is implemented to extract the features from the camera images of the pupa, which enhanced the speed of the feature extraction compared with conventional HOG feature extraction. To enhance the efficiency of the feature set, the HOG feature set is modified by performing LDA dimensionality reduction on each feature block of the HOG feature descriptor. Along with HOG features, weight of the cocoon with pupa is also fused. To obtain the finer feature set, we performed recursive feature elimination (RFE) with different machine learning models. XGBoost ensemble learning obtained the highest results compared to other models. In pursuit of further optimization for the RFEXGBoost model, a novel approach, teaching learning-based population selection genetic algorithm was introduced, and we compared its performance against classical genetic algorithm and grid search for hyper parameter tuning. The advantages of our method have been shown to have a high accuracy rate in sex classification of silkworm pupae, which can help to improve overall efficiency. Unlike traditional destructive methods, our method is non-invasive and does not harm the pupae, allowing them to continue their development and eventual emergence as moths. It is a cost-effective method for pupa sex classification, particularly when compared to other methods such as NIR or PCR. It can also be scaled up or down depending on the size of the production facility, making it a flexible option for the sericulture industry.

## RESULTS

### Performance metrics

In this binary classification task, several metrics including accuracy, precision, recall, F1 score, and the area under the receiver operating characteristic curve (AUC-ROC) are utilized to evaluate the model's efficacy. These performance metrics are calculated using Eqns 1 to 4. The accuracy metric reveals the model's overall ability to correctly distinguish between male and female cocoons. Precision quantifies the model's performance with respect to misclassifications, commonly known as false negatives. Recall assesses the model's efficiency in identifying true positives, avoiding false negatives. The F1 score offers a comprehensive efficiency measure by calculating the weighted average of precision and recall. Lastly, the AUC-ROC serves as an indicator of the model's aptitude for differentiating between the two classes.
(1)

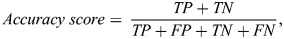

(2)

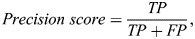

(3)

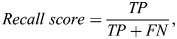

(4)

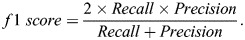


### Data analysis

**Fig. 1.**
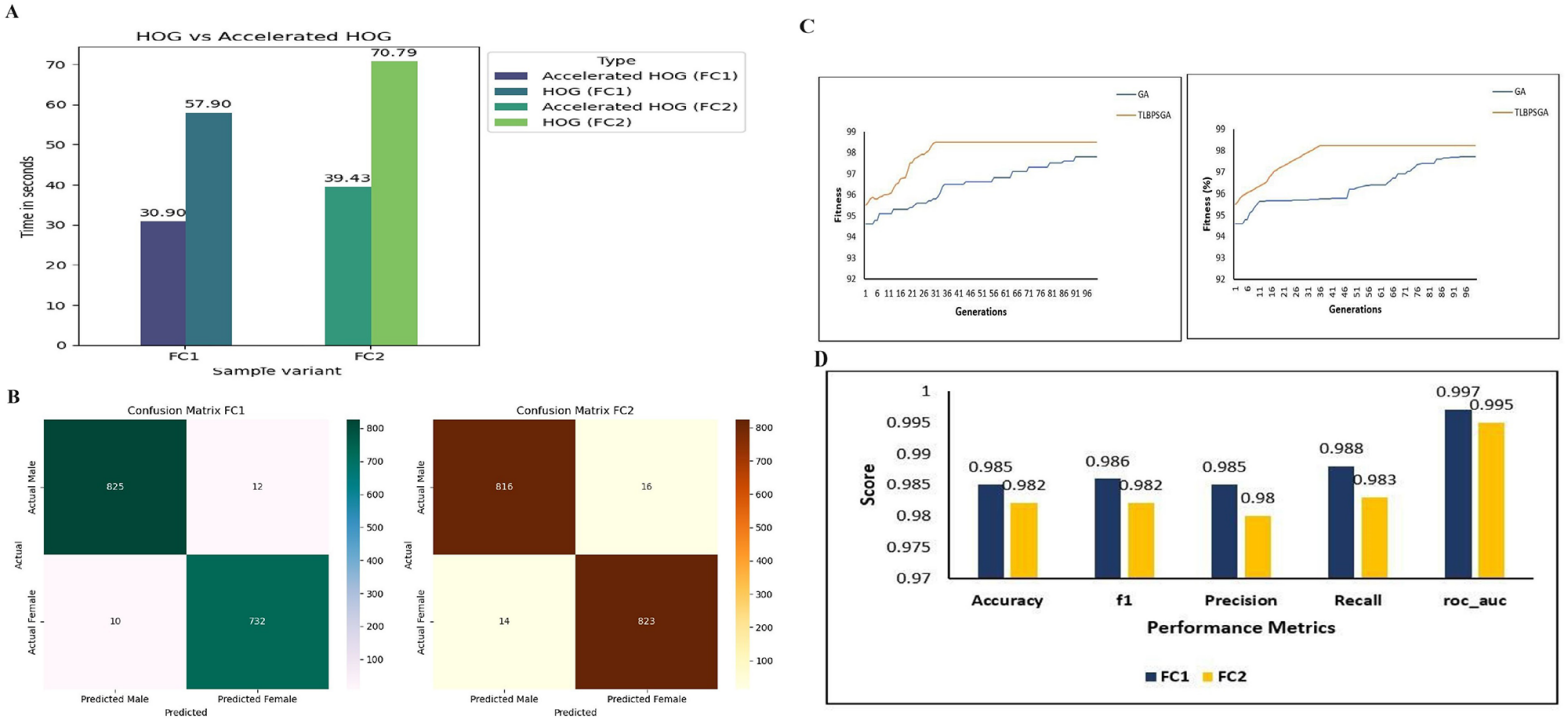
**Performance analysis.** (A) Illustration of HOG versus accelerated HOG. (B) Confusion matrix of FC1 and FC2. (C) Comparison of GA and TLBPSGA RFEXGBoost for FC1 and FC2. (D) Performance analysis based on performance matrices accuracy score, f1 score, precision, recall and ROC-AUC score.

In the initial phase of our proposed methodology, we focused on the critical task of feature extraction, which serves as the foundational step in achieving high classification accuracy. To this end, the HOG descriptor was employed due to its well-established performance in object recognition and image classification tasks. Recognizing the necessity for computational efficiency, we integrated the fast fourier transform (FFT) into the traditional HOG feature extraction process. This amalgamation aimed at accelerating the feature extraction step without compromising the quality of the extracted features. Our empirical results convincingly demonstrated the efficacy of this integration. Specifically, the computational speed for feature extraction was enhanced by approximately 50%, thereby achieving a significant reduction in the computational time required for this critical task. The results are shown in Fig. 1A.

In the subsequent phase of our research, we addressed the issue of high-dimensional feature spaces. We employed linear discriminant analysis (LDA) to perform dimensionality reduction on each feature block. LDA was selected for its well-regarded capability to maximize class separability, thereby retaining the most discriminative features while compressing the feature space. Our results indicate that the application of LDA effectively reduced the dimensionality of each feature block to a manageable size of 105 features. This not only simplified the computational landscape but also optimized the feature set for subsequent classification tasks without sacrificing discriminatory power. To further refine this optimized feature set, we implemented RFE, a technique that systematically eliminates the least important features based on computed feature importance scores.

In the final stage of our investigation, we augmented our feature selection process with a hybrid approach that combined RFE with various classification algorithms. The goal was to evaluate and identify the best performing model for our classification task. Stratified 10-fold cross-validation is employed for analyzing the performance of classification models. This method ensures a more accurate and reliable assessment by maintaining an even distribution of each class across all folds. Each subset of the data is used in a rotation as both the training and testing set, providing a comprehensive evaluation of the model's performance. Performance analysis of FC1 and FC2 are respectively shown in Table 1.

**Table 1.**
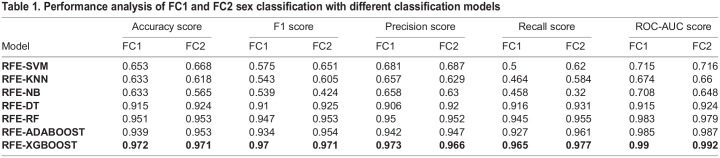
Performance analysis of FC1 and FC2 sex classification with different classification models

The results from this hybrid approach were illuminating. We observed that among the tested algorithms, ensemble methods, specifically the RFE XGBoost model, exhibited superior classification performance. For instance, in classifying FC1 variety, the RFE XGBoost model achieved an accuracy of 97.2%, while for the FC2 variety, it recorded an accuracy of 97.1%.

To further refine the model to improve the performance optimization methods such as grid search, classic genetic algorithm and modified genetic algorithm with teaching learning-based population selection. RFEXGBoost hyperparameters such as learning rate, *n* estimators, maximum depth, minimum child weight, gamma, subsample, column sample by tree were taken for optimization. The range of values used for optimization is given in Table 2. Grid search systematically explores various combinations of hyper parameters for a model. It involves defining a set of possible values or ranges for each hyper parameter of interest and then exhaustively trying all possible combinations of these values. For each combination, the model is trained and evaluated using a cross-validation procedure to assess its performance. The major limitation was that it cannot use a wide range of search space as it is very time-consuming process. Genetic algorithms (GAs) are renowned for their versatility in tackling a vast search space, making them a valuable asset in optimization tasks. They often outpace grid search in terms of convergence speed, but it is worth noting that GAs may require more iterations to reach an optimal solution. However, a promising solution to expedite convergence while reducing the number of iterations is the introduction of a teaching-learning-based population selection genetic algorithm. This innovative approach integrates teaching and learning mechanisms to harness problem-specific insights, steering the optimization process toward promising solution areas with greater efficiency. As a result, this approach not only accelerates convergence but also minimizes the computational effort needed to attain an optimal solution. It strikes a better balance between exploration and exploitation, adapting dynamically to the evolving landscape of complex optimization problems. Table 3 compares the performance of grid search, classical genetic algorithm and teaching learning-based population selection genetic algorithm.

**Table 2.**
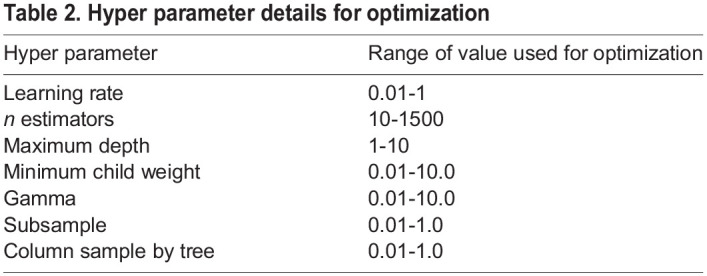
Hyper parameter details for optimization

**Table 3.**
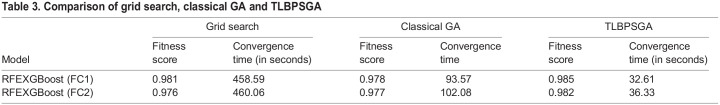
Comparison of grid search, classical GA and TLBPSGA

Analyzing Table 3 reveals that the convergence speed of the TLBPSGA surpasses that of the classic genetic algorithm. TLBPSGA achieves optimal solutions with impressive fitness scores of 0.985 for FC1 and 0.982 for FC2. In this experimental study comprising 100 generations, TLBPSGA accomplishes the optimal fitness score remarkably early, reaching its peak at the 31st generation for FC1 and the 36th generation for FC2. In contrast, the classic genetic algorithm requires significantly more iterations to achieve similar fitness levels, hitting its highest fitness at the 91st generation for FC1 and the 96th generation for FC2.The fitness progression of each generation is visually depicted in the accompanying Fig. 1C, illustrating the notable advantages of TLBPSGA in terms of convergence speed and solution quality. Table 4 shows the optimal hyper parameter obtained for both FC1 and FC2 sex classification. The confusion matrix obtained for FC1 and FC2 silkworm cocoon classification is shown in Fig. 1B. The performance analysis is shown in Fig. 1D.

**Table 4.**
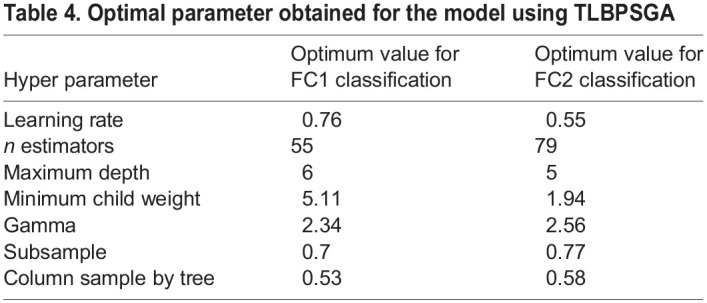
Optimal parameter obtained for the model using TLBPSGA

## DISCUSSION

Our comprehensive study aimed to optimize the feature extraction, feature selection, and classification processes for high-accuracy identification of FC1 and FC2 cocoon varieties. Starting with the integration of FFT into the traditional HOG descriptor, we recorded a dramatic 50% reduction in feature extraction time. As substantiated by the empirical data, the computational speed improved significantly, transforming the feature extraction process from a bottleneck into a much more efficient procedure. This finding underlines the potential of FFT-HOG synergy for a wide range of applications where speed and accuracy are paramount. Moreover, addressing the issue of high-dimensionality, our application of LDA and RFE successfully distilled the feature set down to its most essential elements. This dimensionality reduction not only simplified the computational framework but also enabled us to maintain the discriminatory power of the features, thereby optimizing the feature set for the succeeding classification tasks. It is noteworthy that LDA reduced the feature dimensions to a manageable 105, weight of the cocoon with pupa is integrated to 105 features, while RFE further refined this set, corroborated by the feature importance scores. The final stage involved a hybrid approach that combined RFE with multiple classification algorithms, leading to a series of intriguing observations. Among the ensemble and traditional machine learning methods tested, RFE XGBoost clearly outperformed the others in both FC1 and FC2 categories, achieving accuracies of 97.2% and 97.1%, respectively. These results suggest that ensemble methods are particularly effective in leveraging the quality of the carefully selected and optimized features, especially when the feature selection process is as rigorously executed as in our methodology.

In the pursuit of further optimizing the high-performing RFEXGBoost classifier, introduced a teaching-learning-based population selection genetic algorithm. In contrast to the conventional genetic algorithm, where populations are randomly generated, the proposed approach leveraged a teaching-learning-based optimization process to generate populations. This method led to the acquisition of a more refined population, from which the best individuals were selected for mating in each iteration. This iterative refinement of the population selection process resulted in notable improvements. One significant advantage of this approach is its faster convergence when compared to traditional methods. With fewer generations, the updated genetic algorithm optimization method achieved the convergence of the optimum solution. Following the optimization process, the RFEXGBoost classifier attained an impressive accuracy of 98.5% for FC1 and 98.2% for FC2 sex classification. These accuracy levels represent a substantial improvement over existing methods.

In contextualizing our work within the landscape of existing methods for cocoon classification, a variety of techniques have been explored with varying levels of efficacy, operational suitability, and practical constraints. The exceptional precision of spectral analysis on silkworm pupae is noteworthy, but its practicality is significantly hindered by its destructive nature, as it necessitates the cutting of cocoons. On the other hand, non-invasive techniques like the one proposed by Dai et al. (2021), which employed convolutional neural networks (CNN) on spectral analysis, achieved a commendable 94% accuracy. However, Cai et al. (2014) used X-ray imaging coupled with LDA classification, reporting an accuracy of 93.3%. Despite its effectiveness, this method raises concerns about the potential impact of X-rays on cocoon reproduction and hence, remains less viable for seed production centers. When it comes to cost-effective solutions, camera imaging emerges as a front runner. Mahesh et al. (2017) achieved an accuracy of 91.3% using Neural Networks with features derived from shape and Zernike moments. Joseph Raj et al. (2019) also relied on shape feature extraction, obtaining accuracies ranging from 86.48% to 93.54%. Notably, these studies were conducted with limited sample sizes. Our study significantly extends the existing literature by leveraging a larger dataset, comprising 1579 samples from FC1 variety and 1669 from FC2 variety, totaling 3248 samples. Both FC1 and FC2 are critical for high-yield cocoon production in southern India. The dataset used in our study was validated by the experts in the silkworm seed production center in Palakkad, Kerala, India. These outcomes of the proposed method confirm the efficacy of hybridizing RFE with advanced classification algorithms like XGBoost, particularly in tasks that demand high accuracy. They also validate the effectiveness of our multi-stage optimization strategy, starting from accelerated feature extraction to refined feature selection and finally to hybrid classification and optimization. The proposed non-destructive method for sex classification of silkworm pupae, avoiding cocoon cutting, provides ecological and economic benefits by replacing invasive practices, supporting sustainability, enhancing productivity, and promoting a balanced silkworm life cycle. In the context of seed production, accurate sex classification is pivotal for breeding programs. Being able to classify the sex of pupae without damaging them allows for a more precise and efficient selection process. This in turn enhances the quality of the seed, leading to higher yields and more robust silk production. A more efficient system would reduce the time and labour costs associated with rearing and sexing the pupae, translating into economic benefits for sericulture operations. This insight is also valuable in silk manufacturing centers as the male silkworm cocoons yield a more quality and quantity silk filament than female cocoons (Bu et al., 2022). By differentiating between the filament qualities of male and female cocoons, the silk industry can optimize its processes for higher quality silk output.

## MATERIALS AND METHODS

**Fig. 2.**
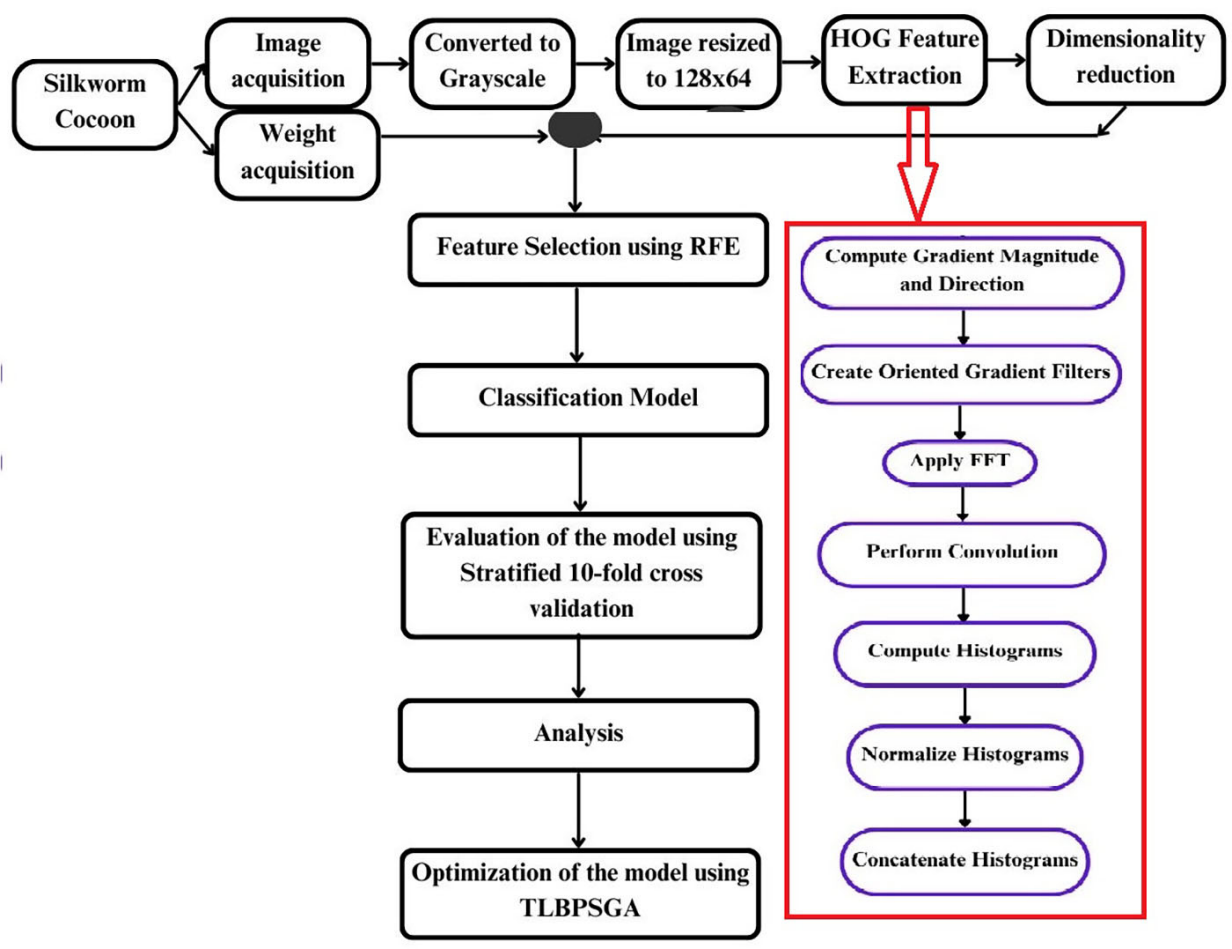
Workflow of the proposed model.

Fig. 2 shows the workflow of the process. The research methodology employed in this study is a sophisticated, multi-stage design to construct a robust and efficient classification model. Initially the FC1 and FC2 cocoon images were acquired to prepare the dataset for the study. Feature extraction is done on the image using fast fourier transform histogram oriented gradient (FFT-HOG). Further dimensionality reduction is performed on each feature block of HOG descriptor to enhance efficiency of the features and then fused weight feature and performed recursive feature elimination with classification models to obtain the highest performing classification model.

### Sample collection

**Fig. 3.**
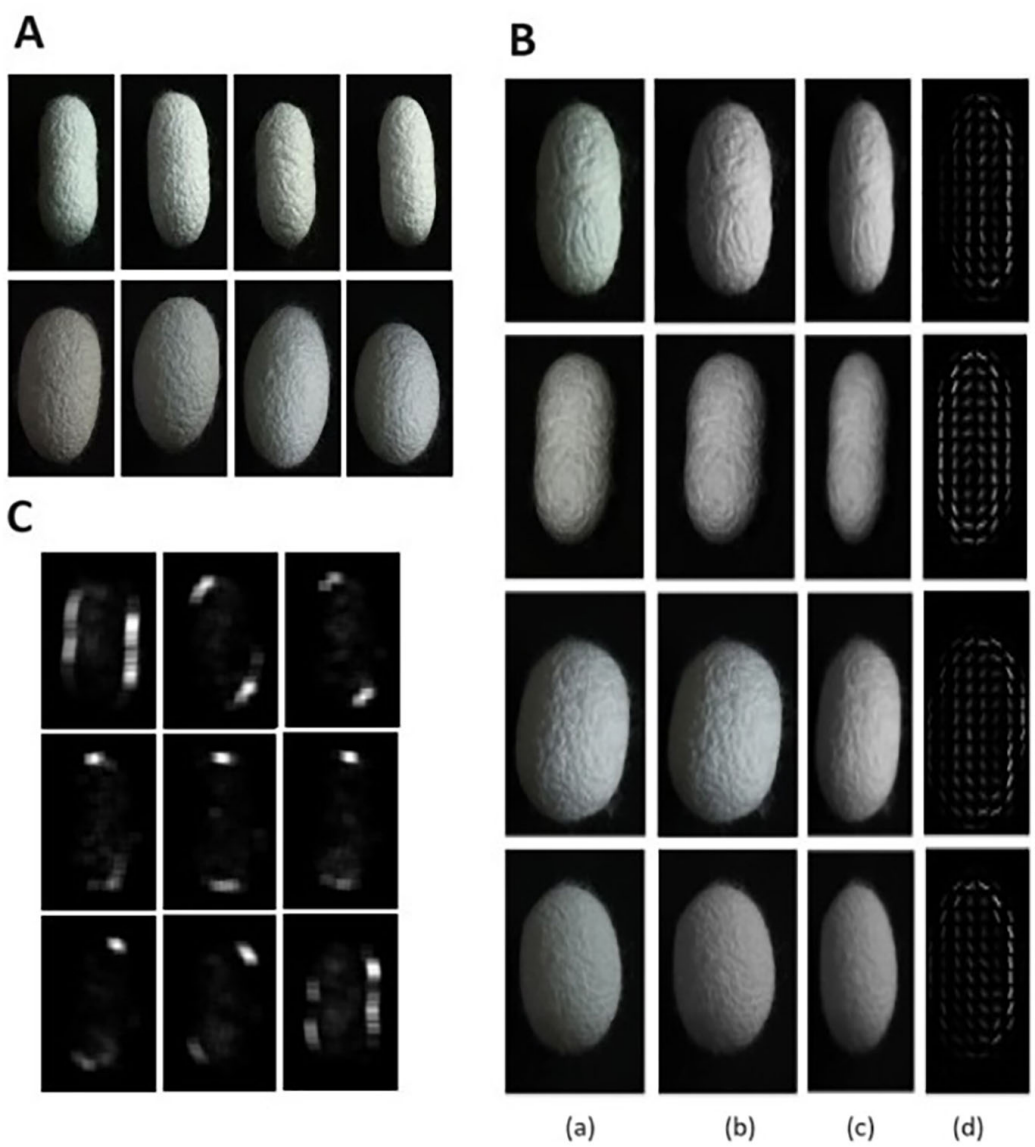
**Image processing on cocoon images.** (A) Sample cocoon images. (B) Visualization of HOG (a) original image (b) greyscale image (c) resized image (d) HOG image. (C) Visualization of FFT conversion of spatial domain data to frequency domain in 9 bin gradient orientation.

In this study, two specific hybrid varieties of silkworm cocoons – FC1 and FC2 – were investigated. The samples were sourced from silkworm rearers authorized by the state sericulture department to rear these particular varieties. The research utilized a dataset comprising 3248 samples, which included 1579 cocoons of the FC1 variety (837 males and 742 females) and 1669 cocoons of the FC2 variety (832 males and 837 females). The validation of the data set was done with the help of experts in the silkworm seed production center in Palakkad, Kerala, India. Sample cocoons are shown in Fig. 3A. The same dataset was used in our previous work (Thomas and Thomas, 2024).

### Feature extraction

Feature extraction is a crucial part in the image classification process. In mulberry silkworm pupa sex classification, HOG features of the images were extracted. The HOG feature descriptor is a technique used in computer vision to represent the distribution of edge directions in an image. It provides a compact representation of local object appearance and shape by quantifying gradient information within localized regions. To enhance the efficiency of the feature extraction process we have combined HOG feature extraction with two dimensional FFT convolution. In this process the images are converted to grayscale and resized to 128×64 for better performance. Gradient magnitude and direction were computed using Eqns 5 and 6:
(5)

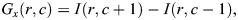

(6)

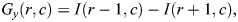
where *G*_*x*_ is the gradient in x direction and *G*_*y*_ is the gradient in the *y* direction, *r* and *c* refer to the rows and columns, respectively. *I* is the intensity value of a pixel. Magnitude and orientation were calculated using Eqns 7 and 8;
(7)

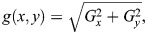

(8)

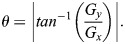
A set of gradient filters were created based on the orientations. Performing convolution in the spatial domain is expensive so to speed up the process gradient magnitude image and gradient filters are converted to frequency domain using Fourier transform then perform the convolution in the frequency domain after this process inverse Fourier transform is applied to convert back to spatial domain, which is shown in Eqn 9. This improves the speed of the feature extraction.
(9)

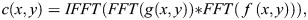
where *c(x, y)* is the result of the convolution at position *(x, y), g(x, y)* is the gradient image and *f(x, y)* is the gradient filter. Now compute the histogram of the filtered image. Normalize the histograms to improve the robustness of the descriptor and then concatenate the histograms to form the final HOG descriptor. Fig. 3B, shows the various stages of HOG feature extraction. Fig. 3C, helps to visualize the frequency domain conversion of the spatial domain data for each of the original 9 bins of gradient orientation.

### Feature engineering

Final HOG descriptor consists of 3780 features, which is very high compared with the number of samples. To improve the efficiency of the classification, process the dataset is divided in to patches of 36 features then applied LDA to reduce the dimensionality to a single component. After performing dimensionality reduction 3780 features were reduced to 105 features.

### Linear discriminant analysis

In classification tasks, LDA is frequently used for reducing dimensionality (Binson et al., 2021e; Anowar et al., 2021). In our research, we initially employed the HOG for feature extraction. Each extracted block comprised 36 features, upon which LDA was subsequently performed for dimensionality reduction. Utilizing Eqns 10 and 11, LDA calculates the mean and covariance matrix for each class. Subsequently, two key matrices are computed, the between-class scatter matrix, gauging the dispersion of mean feature vectors across distinct classes, and the within-class scatter matrix, assessing the dispersion of feature vectors within individual classes.
(10)

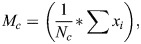

(11)

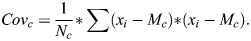


### Weight feature

The weight of each cocoon containing a pupa was meticulously measured using a high-precision single-point load cell. This weight data holds significance as it correlates with the sex of the pupa. Male and female pupae weights are different but there is no significant difference in the weight of male and female cocoons when the pupa is inside the cocoon. We cannot accurately classify them with the weight feature alone. Additionally, this weight-based feature was seamlessly integrated with the extracted HOG feature set with simple concatenation method.

### Recursive feature elimination (RFE)

RFE is a technique commonly used in machine learning and feature selection to improve the performance of models by selecting a subset of the most relevant features from the original feature set. The main idea behind RFE is to iteratively train a model and eliminate the least important features at each iteration until a desired number of features is reached or a certain performance metric is optimized (Senan et al., 2021; Jeon and Oh, 2020).

Initially all the 106 features obtained were taken into consideration then feature importance ranking is computed using mutual information gain. The mutual information gain measures the dependency between two variables. The values of the mutual information gain range between 0 and 1. The maximum mutual information gain and minimum entropy state that the relation between the random variables is high else the relationship is insignificant. The general representation of mutual information gain is as follows:
(12)

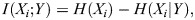
which measures the information gain of the i^th^ feature X_i_ and label Y. Where *H*(*X*_*i*_) is the entropy and *H*(*X*_*i*_|*Y*) is the conditional entropy for *X*_*i*_ given *Y*. Then using different models, recursively the lowest important feature is eliminated, and the performance matrix is analyzed this is repeated until an optimum result is obtained. The details of the classification algorithm.

### Classification algorithms

#### Support vector machine

The SVM serves as a supervised classification method, capable of handling both linearly separable and non-separable data sets. Its primary aim is to identify an optimal separating hyperplane that best distinguishes between classes. To achieve this optimum separation, SVM focuses on maximizing the distance from the hyperplane to the nearest points, known as support vectors, in each class. In essence, the objective is to enlarge the smallest distance between the support vectors and the hyperplane to the maximum extent possible (Chandra and Bedi, 2021; Binson et al., 2021d; Malek et al., 2022). The distance from a data point (x_0_ ,y_0_) to the hyperplane is obtained by Eqn 13:
(13)

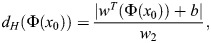
where Φ(*x*_0_) is the point vector, w is the weight vector, b is the bias term and w_2_ is Euclidean norm. Now we need to maximize the minimum distance (distance to the support vector to the hyperplane), which is represented in Eqn 14:
(14)

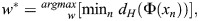
where 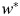 weight vector for the optimum hyperplane, Φ(*x*_*n*_) is the minimum distance data point. The decision is made according to the hyperplane.

#### K-nearest neighbors

The K-nearest neighbors (KNN) algorithm is a form of supervised learning, emphasizing classification based on similarity metrics. In KNN, a data point is assigned to a particular class based on the majority voting mechanism, where votes are sourced from its K closest neighbors in the feature space, as determined by a predefined similarity measure (Zhang et al., 2017; Cover and Hart, 1967). In the context of a dataset, where X represents a feature matrix and Y signifies class labels, KNN aims to estimate the conditional distribution of Y given X. It then categorizes an observation into the class that exhibits the highest conditional probability. Specifically, for a designated positive integer K, the algorithm identifies the K nearest observations to a test data point, denoted as x, and computes the conditional likelihood of x belonging to a certain class, labelled as j using Eqn 15:
(15)

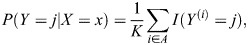
X denotes the matrix of input features, and Y represents the target class labels. The variable j signifies a specific class label, and K indicates the number of closest neighbors considered in the decision-making process. The set A comprises the K closest observations to a point in question. The variable I serves as an indicator for class membership, where a value of 1 means the observation belongs to class j, and a value of 0 means it does not. Choosing a small K results in lower bias but higher variance in the model's predictions, whereas a larger K value leads to reduced variance but increased bias.

#### Naive bayes

Bayes' theorem is applied under the assumption that the predictor variables are conditionally independent. The Naive Bayes algorithm computes the conditional probability of the input variables *x*_i_ for each class variable *y*, and then selects the class that has the highest probability value as the outcome which is represented as follows:
(16)

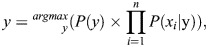
where *P*(*y*) is the class probability, *P*(x|*y*) is the conditional probability. It is assumed that all the input features are normally distributed in Gaussian Naive Bayes. The conditional probability of Gaussian naive Bayes is expressed as follows:
(17)

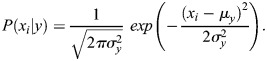
Naive Bayes best works with small training samples and works well with real-world situations (Shaban et al., 2021; Binson et al., 2021b).

#### Decision Tree

The Decision Tree serves as a hierarchical, supervised learning algorithm characterized by a tree-like model of decisions. Within this structure, internal nodes signify decision rules that guide the classification process, while leaf nodes correspond to the final outcomes. The algorithm employs measures of attribute impurity to facilitate node splitting, aiming to maximize information gain for each attribute, thereby ensuring an optimal division at each level of the tree (Charbuty et al., 2021; Cañete-Sifuentes et al., 2021). The entropy and information gain calculation formulas are given below:
(18)

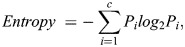
where *P*_*i*_ is the probability of class *i.* c is the total number of classes.
(19)

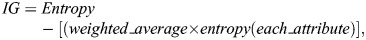
where *IG* is the information gain.

#### Random Forest

Random Forest operates as an ensemble-based classification approach that leverages multiple decision trees for decision making (Parmar et al., 2019; Binson et al., 2021a). The collective outcome, determined by a majority vote among these trees, is what is commonly referred to as bagging. The Random Forest algorithm commences by partitioning the original dataset into a bootstrapped version, wherein samples are randomly drawn to create this new dataset. Decision trees are then constructed using these bootstrapped datasets. During this construction phase, input feature vectors are randomly chosen. Various techniques for this random selection include picking random inputs, generating random combinations, or identifying the most effective feature for splitting. This entire process is iteratively repeated multiple times. The model's performance is subsequently evaluated using samples absent from the original dataset. These evaluation samples are fed into the individual decision trees, and the final class assignment is determined through a majority voting mechanism. The randomization inherent in the algorithm serves to reduce the correlation among the decision trees, thereby enhancing the model's generalization error (G.E.). The equation is given as follows:
(20)

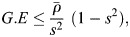
where ρ indicates the mean correlation of decision trees and *s* is the strength of the tree classifier margin.

#### XGBoost

XGBoost (Extreme Gradient Boost) generates a model based on gradient-boosted decision trees introduced by Tianqi Chen (Chen et al., 2015). XGBoost provides a powerful, scalable, and accurate framework for supervised learning tasks such as classification, regression, and ranking. It is particularly well-suited for large datasets and high-dimensional feature spaces, offering features like handling missing values, regularization, and built-in cross validation to enhance model performance (Prabha et al., 2021; Mahesh et al., 2022). In XGBoost, the final model is built based on the loss function calculated from the previous model.
(21)

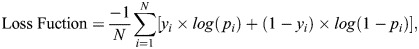
where *N* is the number of samples, *y*_*i*_ is the outcome of the i^th^ sample, *p*_*i*_ is the probability of i^th^ sample. The advantage of using XGBoost is the learning and running time efficiency.

#### AdaBoost

Adaptive boosting (AdaBoost) is an ensemble learning technique aimed at improving the classification performance of weak classifiers. Works by sequentially fitting a series of weak classifiers to weighted versions of the data. After each round, the algorithm updates the weights of incorrectly classified instances, effectively boosting the importance of those instances for subsequent classifiers. The final model is a weighted sum of these weak classifiers, resulting in a strong classifier that often performs significantly better than any individual weak classifier. AdaBoost is particularly effective for high-dimensional data and complex classification tasks. However, it can be sensitive to noisy data and outliers (Wyner et al., 2017; Binson et al., 2021c). AdaBoost gives weights to each training instance in the training dataset to determine its influence on the dataset. Then calculate α, the influence of each decision stumb to the final classifier.
(22)

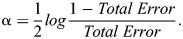
Now new weights are given to the feature instance. If the instance is correctly classified, then α value kept is negative otherwise positive.
(23)

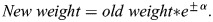


#### Model optimization

To enhance the model's performance, a comprehensive hyper parameter optimization of the chosen XGBoost model was performed. Key hyper parameters identified for fine-tuning included the learning rate, *n* estimators, max depth, min child weight, gamma, subsample, and colsample bytree. For this optimization task, employed the genetic algorithm (GA), a prominent choice among population-based meta heuristic techniques. Notably, to ensure optimal outcomes, incorporated modifications to the classical GA, tailoring it for enhanced efficacy in the context.

### Genetic algorithm

The GA is an optimization technique inspired by natural selection, employing a population-based search strategy guided by the principle of survival of the fittest (Katoch et al., 2021). This algorithm iteratively applies genetic operators to individuals within the population, including chromosome representation, selection, crossover, mutation, and fitness function evaluation (Sabeti et al., 2022). The GA procedure can be summarized as follows: initially, a population of *n* chromosomes is randomly generated. The fitness of each chromosome in the population is assessed. Two chromosomes are selected from the population based on their fitness values. A single-point crossover operator with a specified crossover probability is then applied to the selected chromosomes, resulting in an offspring. Subsequently, a uniform mutation operator is applied to the produced offspring using a specified mutation probability, generating new offspring which is introduced into the new population. These selection, crossover, and mutation operations are iteratively repeated on the current population until the new population is complete. Mathematically, GA dynamically adapts its search process by adjusting the probabilities of crossover and mutation, ultimately converging towards optimal solutions. GA is capable of modifying encoded genes and evaluating multiple individuals, yielding multiple optimal solutions. Consequently, GA exhibits superior global search capabilities. The offspring produced through the crossover of parent chromosomes have the potential to disrupt the favorable genetic schemas of the parent chromosomes. The crossover formula is expressed as follows:
(24)

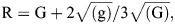
here, g represents the number of generations, and G is the total number of evolutionary generations determined by the population. Eqn 24 demonstrates that the value of R dynamically changes and increases as the number of evolutionary generations rises. In the initial stages of GA, individual similarity is low, and R should be kept low to preserve the integrity of excellent genetic schemas within the population. As evolution progresses, individual similarity increases, and R should subsequently increase. According to the Schema theorem, the original genetic schema must adapt to modified schemas. To maintain diversity within the population, these new schemas are retained during the early stages of evolution. Towards the end of evolution, appropriate schemas are established to safeguard the integrity of excellent genetic schemas, preventing distortion.

**Algorithm 1: Classical GA**

***Input:***

*Population Count, p*
*Maximum iterations, l*

***Output:***

*Best overall solution, B_opt_*

**begin**

*Initiate initial group of p sequences, B_i_ (i=1,2, 3,………p)*
*Set loop counter t=0*
*Determine the fitness score of each member*
*While(t<l)*

*Pick parent sequences from initial group based on their fitness score*

*Conduct Crossover method on chosen pair using crossover likelihood*

*Implement mutation on the descendants using mutation likelihood*

*Substitute the former group with the new generation*

*Increase the counter t by 1*

*End While*

*Return the optimal solution, B_opt_*

**end**

### Teaching-learning-based population selection GA

**Fig. 4.**
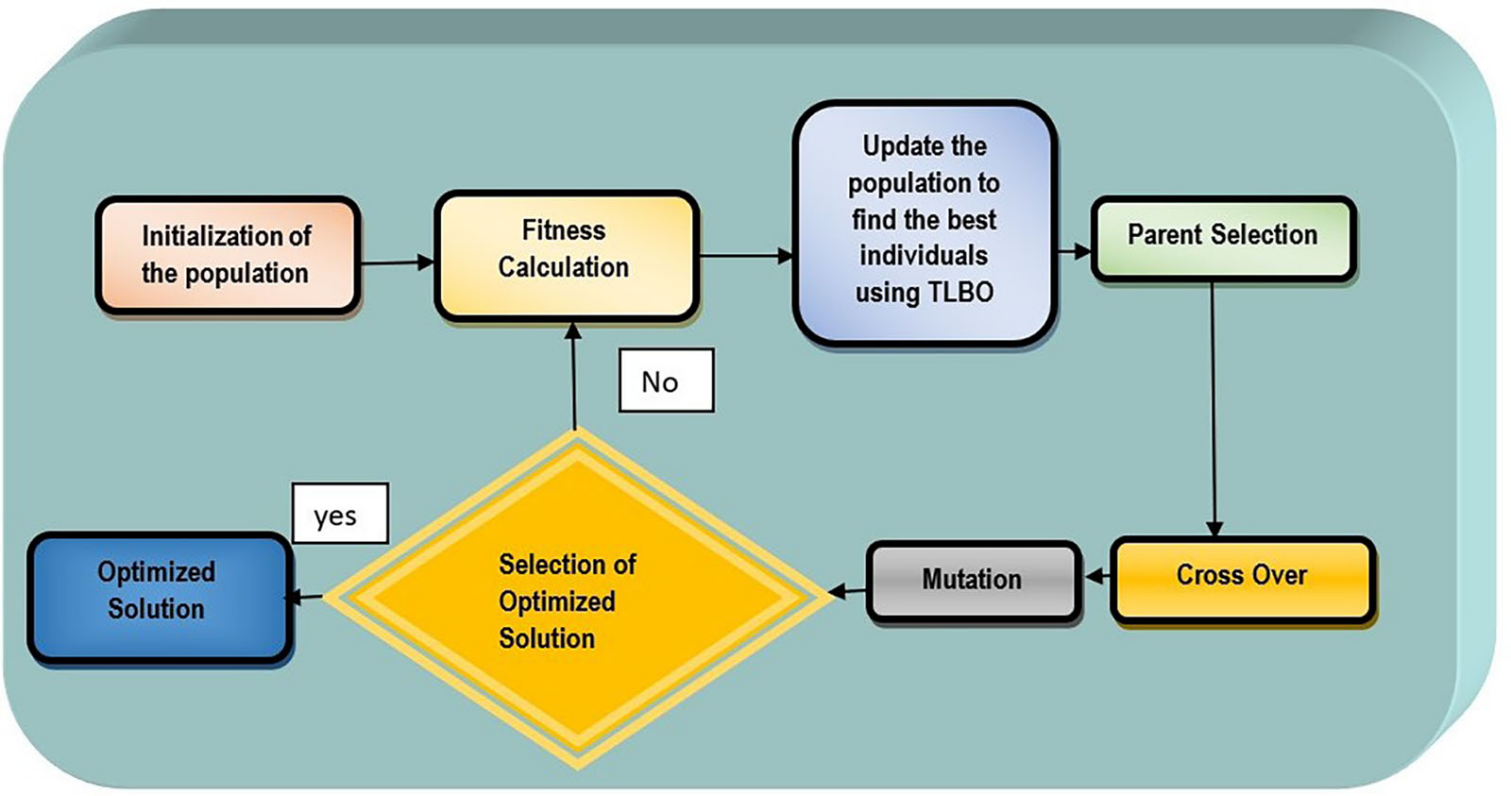
Overview of TLBPSGA.

In contrast to the conventional GA, which typically begins with a random initial population and applies genetic operators to it over several generations to eventually obtain an optimal solution, the proposed approach incorporates a population-refinement process inspired by teaching-learning dynamics (Rao et al., 2011). In this method, the population is initiated with random individuals and then the fitness of each member is calculated. Subsequently, a teaching-learning-based population refinement procedure is performed, which may reduce the population size while aiming to improve the quality of the individuals. For instance, if the initial population comprises 100 individuals, this refinement step may whittle it down to 50 individuals with enhanced characteristics. The primary objective here is to cultivate a population of superior individuals, rather than relying solely on random generation. The population-refinement process consists of two distinct phases: the teaching phase and the learning phase. The teaching phase commences by identifying the individual within the population exhibiting the highest fitness. This distinguished individual assumes the role of the teacher and serves as a reference point for guiding the learning process. The remaining individuals in the population are designated as learners. The enhancement of the learners is facilitated through an iterative update mechanism. Specifically, the solutions of the learners are adjusted using the following equation:
(25)

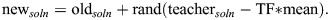
In this equation, the variable rand represents a random factor and TF is the teaching factor. Subsequently, the fitness of the new solution is evaluated for each learner. If the fitness of this new solution surpasses that of the teacher's solution, the teacher is substituted by the improved learner, ensuring that the best solution guides the refinement process. In the subsequent learner phase of the algorithm, a learner is randomly selected from the pool, and this chosen learner engages in interactions with other learners. The key criterion for interaction is rooted in the fitness of the randomly selected learner, which must be superior to that of the currently considered learner. During interaction, solution of the current learner is updated using the following equations:
(26)

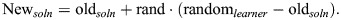
However, if the fitness of the randomly chosen learner does not exceed that of the current learner, the update equation should be adjusted as follows:
(27)

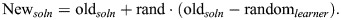
This learner phase contributes to diversifying the population and exploring alternative solutions. In summary, the proposed algorithm adopts a dual-phase approach. It initiates with a teaching phase, where the finest individual guides the learning process, followed by a learner phase that fosters interactions among individuals. By iteratively applying these phases and refining the population, the algorithm aims to elevate the overall solution quality while preserving diversity. Parent selection is carried out from this refined population using the Tournament selection technique, followed by crossover and mutation operations to update the individuals. The iterative process continues until the optimal solution is achieved. The overview of TLBPSGA is depicted in Fig. 4.

**Algorithm 2: Teaching-learning-based population selection genetic algorithm (TLBPSGA)**

***Input:***

*Initial population count, p*
*Maximum iterations, t*

***Output:***

*Optimal solution, B_opt_*

**begin**

*Create an initial set of p members, B_i_ (i=1,2,3,………p)*
*Set loop counter l=0*
*Determine the fitness score of each member*
*While(l<t)*

*//Teaching Phase*

*If fitness(member)==max(fitness)*

*Teacher=member*

*Else*

*Learner=member*

*Iterate through learners and update their solutions using:*

new_solution=old_solution+rand(teacher_solution-Teaching_Factor*mean)

*Evaluate the fitness of the new solution for each learner*

*If fitness(learner_solution)>fitness(Teacher)*

*learner_solution=Teacher*

*//Learner Phase*

*For each learner in the population:*

*Randomly pick another learner*

*If fitness(random_learner)>(current learner)*

*New_solution=old_solution+rand(random_learner-old_solution)*

*Else*

*New_solution=old_solution+rand(old_solution-random_learner)*

*Evaluate the fitness of the new solution and update accordingly*

*//Genetic Operations*

*Use Tournament selection technique for parent selection from the refined population*

*Apply crossover and mutation methods to refine individuals*

*Increment the iteration counter until the optimal solution is found*

*End While*

*Return the best overall solution, B_opt_*

**end**

### Conclusion

Our proposed methodology demonstrates a multi-stage optimization strategy from feature extraction to classification. The FFT-HOG integration offers an expedited feature extraction process, reducing time consumption by about 50% without sacrificing feature quality. Meanwhile, the application of LDA and RFE for dimensionality reduction resulted in an optimized feature set suitable for high-accuracy classification tasks. Importantly, the hybrid RFE XGBoost model emerged as the most effective classification algorithm, thus validating the robustness of our entire methodological pipeline. TLBPSGA enhanced the efficiency of the RFE XGBoost Model, outperforming traditional genetic algorithms in convergence time and achieved impressive accuracy levels, 98.5% for FC1 and 98.2% for FC2. The study contributes to the literature by providing a well-vetted, efficient, and high-performing method for FC1 and FC2 cocoon variety classification. This research holds promising implications not only for the scientific community engaged in machine learning and computer vision but also for practical, real-world applications in agriculture and silk-production.
